# Glycated Haemoglobin Is Inversely Related to Serum Vitamin D Levels in Type 2 Diabetic Patients

**DOI:** 10.1371/journal.pone.0082733

**Published:** 2013-12-16

**Authors:** Giacomo Zoppini, Anna Galletti, Giovanni Targher, Corinna Brangani, Isabella Pichiri, Carlo Negri, Vincenzo Stoico, Vittorio Cacciatori, Enzo Bonora

**Affiliations:** Section of Endocrinology, Diabetes and Metabolism, Department of Medicine, Azienda Ospedaliera Universitaria Integrata, University of Verona, Verona, Italy; University of Padova, Medical School, Italy

## Abstract

**Objective:**

A correlation between glucose control and 25(OH)D metabolism has been suggested by previous studies. However, this correlation has not yet been evaluated considering the impact of chronic complications of type 2 diabetes, especially the presence of nephropathy. Thus, the aim of this study was to determine the correlation between A1C and 25(OH)D in a well characterized cohort of type 2 diabetic patients.

**Research Design and Methods:**

We cross-sectionally examined the association between A1C and serum 25(OH) D in 715 type 2 diabetic patients attending our clinic during the years 2011–2012. The average age was 68±12 years (range 26–94 years). The relation between A1C and serum 25(OH)D levels was modelled by multiple linear regression analyses.

**Results:**

Serum 25(OH)D levels were inversely associated with A1C levels (r = −0.116, p = .003). This relation maintains its independence in the multivariate analysis after adjusting for age, sex, A1C, BMI, treatment and duration of diabetes and nephropathy.

**Conclusions:**

In type 2 diabetic patients, high A1C levels are associated with low concentrations of serum 25(OH)D independently of duration of diabetes, diabetic treatment and nephropathy. Future studies are needed to clarify the biological relation between glucose control and vitamin D metabolism in type 2 diabetes.

## Introduction

Vitamin D is a fundamental micronutrient with major implications for human health [1]. Its insufficiency has been reported to be a quite common finding in type 2 diabetic patients [2]–[4]. Human and animal studies have shown a negative correlation between serum levels of vitamin D and both serum glucose and insulin levels, whereas the correlation with insulin sensitivity was positive [5], [6]. In diabetic and non diabetic subjects, a significant inverse relationship between glycated haemoglobin (A1C) and serum 25(OH)D levels has been observed [7]–[10]. Vitamin D may improve glucose-stimulated insulin secretion in pancreatic β-cells [11], enhance glucose and lipid metabolism in skeletal muscle [12], [13], and ameliorate systemic inflammation [14]. Most, but not all, patients with T2D or glucose intolerance may have lower serum 25(OH)D levels when compared to healthy control subjects [15]. Interestingly, a recent study found that serum 25(OH)D levels increased after the correction of acute hyperglycemia [16], suggesting a bidirectional biologic relation between blood glucose levels and 25(OH)D metabolism. In order to pursue this hypothesis we believe that it is important to determine whether the correlation between A1C and 25(OH)D is independent when adjusted for influential variables, such as duration of diabetes or nephropathy.

Therefore, the aim of the present study was to investigate the independence of the relation between A1C and serum 25(OH)D in an ample cohort of ambulatory type 2 diabetic.

## Research Design and Methods

For this observational analysis, the electronic records of all type 2 diabetic outpatients attending our clinic during the years 2011–2012, were analyzed. A sample of 715 type 2 diabetic subjects who had a serum 25(OH)D measurement available was examined. The average age of patients was 68±12 years (range 26–94 years) with a 61% of women. Patients were classified as type 2 diabetics when the diagnosis had been made after 35 years of age, irrespective of treatment, or, irrespective of age of diagnosis, if treated with diet and/or hypoglycaemic agents. None of the subjects under study was on chronic dialysis or affected by severe liver disease. The study protocol was approved by local ethics committee “Azienda Ospedaliera Universitaria Integrata of Verona”. The informed consent requirement for this study was exempted by the ethics committee because researchers accessed only retrospectively to a de-identified database for analysis purposes. Body mass index (BMI) was calculated by dividing weight in kilograms by the square of height in meters. Blood pressure was measured with a standard mercury manometer. Venous blood was drawn in the morning (8:00–8:30 AM) after an overnight fast in all subjects. Biochemical measurements were determined by standard procedures (DAX 96; Bayer Diagnostics, Milan, Italy). Hemoglobin A1c was measured, according to the IFCC standards, by automated high-performance liquid chromatography analyzer (Bio_Rad Diamat, Milan, Italy); the upper limit of normal for our laboratory was 5.8%. The concentrations of total serum vitamin D (25(OH)D along with the others hydroxylated metabolites of vitamin D) were determined by chemiluminescence (CLIA, DiaSorin Liaison, Stillwater, USA) with a coefficient of variation of 8.6%. Patients were considered to have arterial hypertension if their blood pressure values were ≥ 140/90 mmHg or they were taking anti-hypertensive agents. Glomerular filtration rate (GFR) was estimated from the four-variable Modification of Diet in Renal Disease study equation [17]. Urinary albumin excretion rate was measured from a 24-h urine sample using an immunonephelometric method. The presence of abnormal albuminuria (defined as albumin excretion rate > 30 mg/day) was confirmed in at least two out of three consecutive urine samples [17]. Nephropathy was considered absent (0) when no albuminuria and eGFR ≥ 60 ml/min.1.73 m^2^ were reported, or present (1) when albuminuria (micro or macroalbuminuria) or eGFR < 60 ml/min.1.73 m^2^ were reported. Treatment was categorized in diet/oral agents/insulin alone or associated to oral agents.

### Statistical analysis

Data are presented as means±SD or proportions. Skewed variables were logarithmically transformed to improve normality prior to analysis. The Student's t-test and the chi-squared test with Yates correction for continuity were used to analyze the differences among the characteristics of participants. Univariate analysis (Pearson's correlation coefficient or Spearman rank analysis) was performed to look for correlations between variables. Linear regression models were done to approach modelling the relationship between the natural logarithm of serum 25(OH)D level and covariates. These covariates were chosen as potential confounding factors on the basis of their significance in the univariate analysis or on the basis of their biological plausibility (i.e. the presence of nephropathy). All covariates were simultaneously included in the multivariate regression models (forced-entry). Sex, diabetes treatment and nephropathy were included as dummy variables. P<0.05 was accepted as statistically significant.

## Results

**Figure 1:**
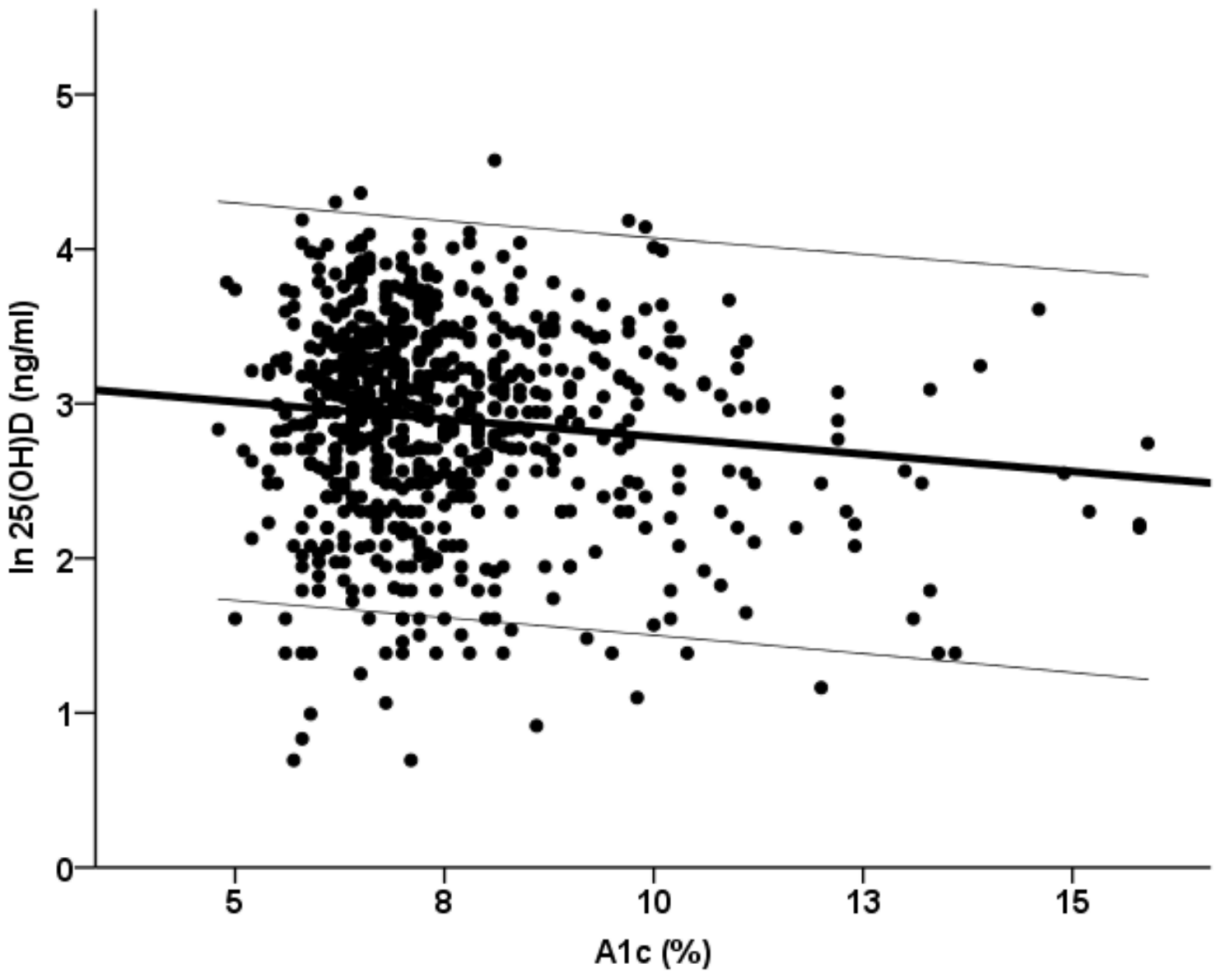
Dispersion graph of the relation between serum ln25(OH)D and glycated haemoglobin in 715 type 2 diabetic subjects. The regression with the 95% CI lines are shown.

In this cohort of 715 ambulatory type 2 diabetic patients, we found a significant inverse correlation between A1c and serum ln25(OH)D (r = −0.116, p = .003) (Figure 1), while no significant correlation was detected with fasting plasma glucose (r = −0.066, p = .122). The dispersion graph shown in Figure 1 suggested an accentuated decline in the concentration of serum 25(OH)D at higher levels of A1C. In order to further characterize the relation between these two variables, we categorized A1C in deciles. Serum 25(OH)D levels started declining in the last two deciles, corresponding to a A1C level of about 9%: this inflection point might suggest a threshold. Consequently, patients were grouped accordingly: glycated haemoglobin > 9% (a clear poor metabolic control) or a A1c below 9%. In Table 1, the main clinical characteristics of subjects stratified according to A1C of 9%, are reported. Subjects with poor glycemic control, as expected, had a significant lower 25(OH)D concentrations, while BMI tended to be higher. Patients with higher levels of A1C also showed a significantly higher prevalence of nephropathy and were more frequently on insulin treatment. To assess the independence of the relation between A1C and serum 25(OH)D, different models of multivariate linear regression were performed. As shown in Table 2, the inverse correlation between A1C and the natural logarithm of serum 25(OH)D levels was significant when adjusted for age and BMI (model 1; R^2^ 9.1%). Even after adding variables more related to diabetes severity, the relation between A1C and the natural logarithm of 25(OH)D maintained the statistical significance (model 2; R^2^ 10.5%). Other variables significantly associated with serum 25(OH)D levels were age, BMI, duration of diabetes and nephropathy. A1c levels were still significantly and inversely associated to serum 25 (OH)D when in the model was added retinopathy (A1C, standardized β coefficient – 0.107, p = 0.026).

**Table 1:** Clinical characteristics of type 2 diabetic patients in relation to glycated hemoglobin above and below 9%.

	HbA1c<9%	HbA1c ≥ 9%	p
	n = 599	n = 116	
Age (years)	69±12	67±12	.150
Sex (% M)	39%	39%	.502
25(OH)D (ng/ml)	22.5±13.2	18.9±12.5	.008
BMI (Kg/m^2^)	31.1±7.6	32.1±8.5	.057
Duration of diabetes (years)	10.5±10.0	10.4±9,6	.958
SBP (mmHg)	138.2±18.3	138.6±21.9	.867
DBP (mmHg)	80.5±10.6	80.1±11.8	.753
A1C (%)	6.9±0.8	10.6±1,6	ND
eGFR (ml/min.1.73 m^2^)	74.4±19.6	75.4±21	.683
**Diabetes treatment**			<.001
Diet	13%	0%	
Oral agents (%)	62%	35%	
Insulin alone or in combination (%)	25%	65%	
Nephropathy (%)	43.7%	58.2%	.005

= 715. Cohort size: n

±SD or percentages. ND =  not determined. A1C: glycated haemoglobin. BMI: body mass index. SBP: systolic blood pressure. DBP: diastolic blood pressure. EGFR: estimated glomerular filtration rate. Data are expressed as means

**Table 2:** Linear multivariate models with ln 25(OH)D levels as dependent variable.

	Model 1		Model 2	
	Stand.β coeff.	p	Stand.β coeff.	p
A1C (%)	−.115	.002	−.109	.014
Age (years)	.183	<.001	.218	<.001
Sex	−027	.473	−.017	.687
BMI (Kg/m^2^)	−.131	.002	−.140	.001
Treatment			.003	.954
Nephropathy			−.092	.038
Duration of diabetes (years)			−.089	.050
R^2^	9.1			10.5

= 715. Cohort size: n

β coeff: standardized β coefficient. A1C: glycated haemoglobin. BMI: body mass index. Stand.

## Discussion

In the present study we confirmed a significant inverse correlation between A1C and serum 25(OH)D levels, a good indicator of vitamin D status. We also reported that this significant inverse relation persisted in the multivariate analyses: A1C was a significant predictor of serum 25(OH)D levels independently of age, BMI, duration of diabetes and nephropathy. There was no correlation between fasting glycemia and 25(OH)D, thus suggesting that glucose control over time, as reflected by the levels of A1C, is probably more important. This study confirms in part previous results and extends our understanding since we demonstrated that the correlation between A1C and serum 25(OH)D levels is independent after adjusting for variables indicative of the severity of diabetes, such as duration, treatment and complications of the disease itself. Since this correlation can be bidirectional, we believe that it is important to assess its independence from confounders related to the severity of diabetes. Previous studies have observed a significant inverse correlation between A1C and serum 25(OH)D levels both in non diabetic and in diabetic subjects [7]–[10]. A study reported a significant inverse correlation only in diabetic women [8]. However, one study did not find a significant correlation in diabetic patients who were microalbuminuric and with a high cardiovascular risk profile [18]. Other authors failed to find a significant impact on A1C levels in subjects with low concentrations of serum 25(OH)D levels (less than 20 ng/ml) [19]. Even the supplementation with vitamin D failed to affect positively the glycemic control [20], [21].

However, no studies have yet extensively evaluated the relation between these two variables by taking into account the characteristics of diabetes severity, such as duration of the disease, treatment and the presence of complications that can partly explain the discrepancy observed among the studies.

Indeed, we observed a significant inverse correlation between HbA1c and serum 25(OH)D levels that persisted in the multivariate analysis, thus suggesting a possible connection between glycemic control and vitamin D metabolism.

Glycemic control may affect serum 25(OH)D levels by different mechanisms. A worse glycemic control can simply be associated with a poorer diet habit and/or to a lower exposure to sunlight. However, an alternative and potentially interesting explanation could be that a poor chronic glycemic control directly affects vitamin D metabolism. The first hydroxylation process takes place in the liver and forms 25-hydroxyvitamin D3 (25(OH)D3), while the second hydroxylation step, which produces the final active metabolite, occurs predominantly in the kidney. These reactions are brought about by 25-hydroxylase in the liver and 1a-hydroxylase in the kidney, and they belong to the cytochrome P450–dependent steroid hydroxylases [5]. Two enzymes in the liver, one in microsomal fraction and the other in mitochondria, catalyze the 25-hydroxylation of vitamin D [22]. A study carried out on rats with experimental diabetes found that the low levels of serum 25(OH)D found in diabetic animals could be attributed to a reduction of 25-hydroxylase activity in the liver [23]. In addition, the observation that the correction of acute hyperglycemia determined an increase in serum 25(OH)D levels [16] could suggest that hyperglycemia may interfere with the activity of the 25-hydroxylase. However, no direct evidence of a possible effect of hyperglycemia on 25-hydroxylase has been provided yet, an issue that only specifically designed studies can address. Nevertheless, we believe that our results, if confirmed, can have clinical implications. The supplementations of vitamin D in diabetic patients in poor metabolic control (i.e. A1C>9%) can result inefficacious due to the low activity of 25-hydroxylase in the liver.

The current analysis has limitations: it is a cross-sectional study based on hospital data thus not allowing to infer causality; moreover we did not have data on vitamin D supplementations or sun exposures. Moreover, as with all observational studies, hidden biases or inability to account for all factors related to vitamin D metabolism might have limited our multivariate approach. Nevertheless, despite these limitations, the study has some strength: a large sample of well characterized type 2 diabetic patients and the completeness of the database.

In conclusion, high A1C concentrations are associated with a low concentration of serum 25(OH)D in type 2 diabetic patients independently of the duration of diabetes, diabetes treatment and nephropathy. Future studies are needed to elucidate the biological relation between glycemic control and vitamin D metabolism in diabetes.
